# Oocyte donors’ awareness on donation procedure and risks: A call for developing guidelines for health tourism in oocyte donation programmes

**DOI:** 10.4274/jtgga.galenos.2018.2018.0110

**Published:** 2019-11-28

**Authors:** Pınar Tulay, Okan Atılan

**Affiliations:** 1 Department of Medical Genetics, Near East University Faculty of Medicine, Nicosia, Cyprus; 2 Near East University, Research Centre of Experimental Health Sciences, Nicosia, Cyprus; 3 Department of Medical Biology and Genetics, Near East University Institute of Health Sciences, Nicosia, Cyprus

**Keywords:** Oocyte donation, donor programme, donors, ethics

## Abstract

**Objective:**

In the recent years, oocyte donation programmes have widely spread worldwide becoming the drive of health tourism. In some countries, donation programmes are tightly regulated, whereas in others, the guidelines or regulations are not well defined. To evaluate donors’ awareness of the donation programmes and the ethical consequences in enrolling these programmes.

**Material and Methods:**

A detailed questionnaire-based survey was conducted to evaluate the donors’ main drive to get involved in the donation programme and the donor’s knowledge and awareness of risk factors.

**Results:**

The majority of the donors (70%) were undergoing donation programmes for financial gains through compensation. The donors were especially not aware of the long-term medical risks and the possibility of identity exposure through genetic screening.

**Conclusion:**

The main duty of health professionals is to counsel donors about the basic procedures and any possible problems they may face during the donation programmes. Reimbursement of oocyte donors is a slippery slope in oocyte donation programmes. High compensation may make women think that donation is a profession without considering possible risks. Furthermore, with the wider use of direct-to-consumer genetic testing, and genetic anonymity may be at risk, thus the donors have to be counselled properly. Therefore, in this era of health tourism, it is crucial to set up well-defined counselling bodies in all oocyte donation centres and enable donors to make an informed choice in becoming oocyte donors.

## Introduction

Third-party reproduction has become one of the widely used fertility treatments that involve use of gametes or embryos. With the improvements in oocyte cryopreservation techniques, a new era of health tourism has been initiated. The first oocyte donation was performed in 1983 in Austria and since then it has become a part of routine assisted reproductive technology (ART) treatments (1). Thousands of oocyte donations have been applied throughout the world resulting in thousands of births (2). The main drive of oocyte donations is the inability of females to get pregnant using their own gametes due to poor oocyte quality after several failed in vitro fertilization (IVF) attempts or low/absent ovarian reserve because of advanced maternal age or premature ovarian failure. Oocyte donation can also be offered to woman with a heritable genetic disease to prevent the transmission of the disorder to the next generation, though preimplantation genetic diagnosis is usually preferred with no history of infertility. Least commonly, oocyte donations can be offered to same-sex male couples in adjunct to surrogacy.

Reproductive cells, especially oocyte cells, are supplied by a limited number of donors, similar to other organ and tissue donations. Controlled ovarian hyperstimulation (COH) protocols have to be applied to all patients undergoing ART treatments. These protocols have been long revised and although more studies are being performed to enhance them, they are very standardised. However, there is always a worry that higher doses of drugs may be used to obtain more oocytes from the donors because the number of patients seeking donor oocytes is always higher than the number of donors. The short- and long-term medical risks of COH have been investigated with a limited number of studies. These studies have suggested that there is an increased risk of early menopause and ovarian cancers (3). Furthermore, donors may undergo multiple COH cycles, especially in countries where there are no regulations.

The oocyte donation process is considered to be a slippery slope because it does not benefit the donor directly (4,5,6,7). The majority of oocyte banks provide financial compensation that raises substantial ethical concerns on the quality of informed consent (8) with the exploitation of oocyte donors (9,10,11). There has always been considerable concern on the undue inducement that affects the judgement of the women’s ability to rationalise and evaluate the burdens and risks of participating in the oocyte donation programme (12). There have been long-running discussions regarding females with low socioeconomic status being exploited by donation programmes (13). One of the other concerning areas in the donation programmes is the sufficiency of counselling provided to the donors.

In addition to these objective and subjective matters of oocyte donations, concerns of elimination of anonymity have arisen due to the direct to consumer (DTC) genetic testing. Throughout the world, oocyte banks have been established with stored donor oocytes of mostly anonymous donations (2). In recent years, more and more people are taking DTC genetic testing without even consulting a physician. Mostly, individuals are taking these tests to uncover their ancestry or to learn about their possible health issues (14). However, a number of cases have been reported where the child is seeking the biologic parent using the results of DTC genetic testing (1,15). Furthermore, we are entering into the era of personalised medicine, where genomic databases storing patients’ information are being formed to provide better individualised medical care. A person’s genome can be sequenced even before they are born. It is still not exactly clear as to whom this data will be available and under what circumstances. However, it is highly unrealistic to expect genomic anonymity in this genomic era.

Although in some countries the donation programmes are tightly regulated; in some, there are no strict guidelines or regulations (1). This introduces many issues in such countries where oocyte donations become the drive of health tourism. A scarce number of studies have investigated this; however, many factors, such as the demographics, education level, and socioeconomic status, may alter the reasons of the involvement in oocyte donation programmes.

The aim of this study was to evaluate the ethical aspects of oocyte donations, the counselling services provided to donors, and donors’ awareness of the consequences in undergoing donation programmes. More specifically; we intended to determine the counselling quality before the treatment, and the depth of the information provided by health specialists. We aimed to investigate the donors’ knowledge regarding the fate of the donated oocytes, the short- and long-term medical risks, and the ethical implications of the donation. Furthermore, we investigated the reasons for the donors to get involved in the oocyte donation programmes.

## Material and Methods

### Ethical approval

A total of 50 donors volunteered for the study. Ethical approval was granted by the Near East University Ethics Committee (Project number: YDU/2018/58-604) prior to commencement of the study and informed consent was obtained from all participants. A questionnaire-based survey was used as an evaluation method. A thorough literature review was performed to prepare the questionnaire using a Likert scale (Appendix 1). The questions were mainly focused on the evaluation of the reasons for involvement in the donation programme, donor’s awareness, and knowledge of risk factors and genetic screening.

### Study population

The participation was anonymous and voluntary. The women included in this study were recruited as oocyte donors in a private IVF clinic as part of a donation programme. They had to meet specific requirements to become a donor and these requirements also determined their suitability to be included in this study. Donors had to be aged between 18 and 32 years and they had to be screened negative for sexually transmitted infections including human immunodeficiency virus, cytomegalovirus, and hepatitis B and C. They had to have a normal physical and gynaecologic examination and no familial history of congenital malformations or hereditary diseases. A good physical and mental health were also required to be an eligible donor. The potential donors meeting these requirements were assessed by the gynaecologist and counselled by the same gynaecologist and/or IVF nurse.

### Subjective and objective questionnaires

The questionnaire was presented to the participants after they had been through the oocyte collection procedure and were fully recovered from anaesthesia. The survey was performed by an experienced nurse and/or an experienced embryologist. The demographic characteristics were reported for all the donors. These included age, level of education, marital status, reasons of involvement in the donation programme, and socioeconomic status. Subjective questionnaires were designed to interrogate the perceived understanding of the oocyte donations. These involved questions to rate how well the donors understood the oocyte donation process. The objective questionnaires involved interrogating the objective understanding, including the understanding of matters, such as “There is a risk that I can become pregnant naturally if I engage in a sexual relationship during the donation process” or “I can change my mind about donating my oocytes”. Open-ended questions were also recorded to specify the motivation of the involvement in the donation programme and to identify what had driven them to donate their oocytes. The answers were mainly grouped as, financial gain, helping couples who cannot get pregnant using the female partner’s oocytes, and other reasons, such as knowing the person who needed the oocytes.

### Statistical analysis

Fisher’s exact test was performed and a two-tailed p value of less than 0.05 was considered to be statistically significant.

## Results

**Figure 1:**
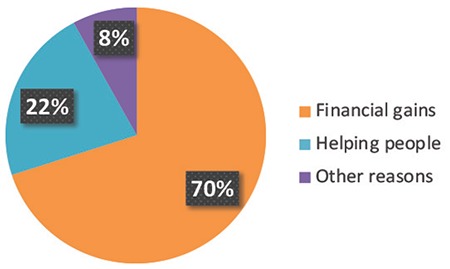
Pie chart representing the main reasons for donation in our cohort of oocyte donors

In this study, a total of 50 donors volunteered to perform the survey investigating the main drive in the involvement of oocyte donation programmes and the awareness of the procedure and risks. Our cohort of oocyte donors indicated that the main reason for donating their oocytes was due to financial reasons. Seventy percent (n=35) of the donors underwent this programme to benefit from financial gains, 22% (n=11) stated that they had always wanted to help someone going through infertility problems and were donating mainly for altruistic reasons. The remaining 8% (n=4) donated oocytes for other reasons or did not want to disclose their particular reason (Figure 1).

These donors were further questioned regarding the number donation programmes they were involved in. The majority of the donors (80%, n=40) had donated their oocytes multiple times. Overall, 12.5% (n=5) did not want to reveal the number of donations they had previously been through. Sixty-eight percent (n=38) of the donors had undergone an average of 4.76 previous oocyte donations ranging from 2 to 9 COH cycles. Twenty-one percent (n=7) of the donors further specified that they had donated oocytes in different ART centres.

**Figure 2:**
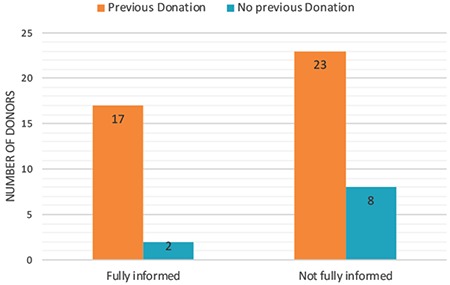
Bar chart representing counselling provided to the donors about the procedures and risks of donations

The final part of this study investigated the knowledge of donors on: (i) the procedure before the initiation of donation, (ii) the procedure during/after donation, (iii) the fate of oocytes after donation, (iv) the short-term medical risks of donation, (v) the long-term medical risks of donation, and (vi) the ethical implications of donation, such as the possibility of identity disclosure through genetic testing. Overall, only 38% (n=19) of donors were fully aware of all the procedures, the fate of the oocytes, and the medical risks. Of these well-informed donors, only 4% (n=2) were first-time donors (Figure 2). More than half of the donors with previous experience in donations (57.5%, n=23) were not fully aware of what the procedures were. The average rate of knowledge among donors with previous donation history was 88% (n=35), where the average rate of knowledge of first-time donors was 71% (n=7). The least informed donor was a first-time donor with a knowledge rate of 29%. Donors were relatively well informed about the fate of the oocytes before and during the donation procedure. Overall, the donors were least informed about the long-term medical risks, in which 52% (n=26) stated that they had not been informed about these risks at all. Furthermore, they were not well informed about the short-term medical risks such as risks associated with anaesthesia, infection/bleeding after oocyte collection, and bruising from injections/withdrawal of blood. Twenty-four percent (n=12) of the donors also stated that they were not informed about the possibility of identification after genetic testing. All donors were aware of the risk of ovarian hyperstimulation syndrome (OHSS); however, only four percent were aware of the risk of pregnancy during treatment cycle in case of unprotected sexual intercourse.

**Figure 3:**
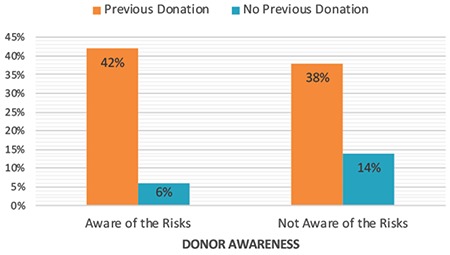
Bar chart representing the awareness of donors about the procedures and risks of donations

To investigate the knowledge of the donors with previous donations, 2×2 contingency tables were formed. The association between previous donation and the awareness was statistically significant (p<0.0001) for the first four categories; (i) the procedure before initiation of donation, (ii) the procedure during/after donation, (iii) the fate of oocytes after donation, (iv) the short-term medical risks of donation. The association between previous donation and awareness of the possibility of identity disclosure through genetic testing was also statistically significant (p<0.05). However, donors with a previous donation history were equally as unaware as first-time donors about the long-term medical risks of donation (p>0.05, Figure 3).

## Discussion

Ethical and psychological aspects of oocyte donation programmes present distinct challenges in reproductive medicine. Donating oocytes is risky in terms of the actual medical procedure of obtaining oocytes and in terms of the short- and long-term health risks that the procedure and the use of (repeated) supra-physiologic hormones might have on the donors. Moreover, donors are often faced with the ethical dilemma of how their reproductive cells might be used and will usually have no hereditary autonomy after the donation procedure. Depending on the demographics of women participating in donation programmes, the reasons of becoming a donor show variability. Although some donors volunteer to become a donor with no reimbursement, some receive different amounts of compensation introducing issues on donor exploitation. The aim of this study was to investigate the drive of becoming oocyte donors and to assess how well these donors were informed about the stages of the procedure and the associated risks.

One of the most important aspects of donation programmes is sufficient counselling. The results of this study showed that the counselling on the most basic procedure, possible short- and long-term medical risks were not reasonable in North Cyprus. This finding is particularly worrying because it implies that oocyte donors do not give true informed consent. This was in agreement with previously published studies, in which only 34% of the donors were aware of the COH procedure, only 20% were aware of the risk of bleeding or infection, and only 15% of ovarian torsion or damage (14). Therefore, it is crucial to develop counselling services to improve the subjective and objective understanding for the oocyte donors. Although counselling methods show variations in different ART clinics, they have the responsibility to ensure that donors are well informed about the procedures and the risks. One-on-one discussion was suggested to be the only intervention to improve donor perception on the subject (16). Therefore, it is a possibility that clinics may adapt their counselling programme with a one-on-one discussion. Another important part of counselling is to cover the concept of anonymity. Both the parents using the donated oocytes and the donors have to be fully informed that the DNA obtained from the children reveals information on the biological parents. The anonymity in the genomic era where DTC genetic testing is becoming more and more available will be eliminated rapidly. The challenges faced due to compensation combined with limited information given might make donating oocytes seem a risk-free, comfortable way of earning money.

One of the greatest societal and ethical concerns of oocyte donations is the amount of reimbursement to avoid donor exploitation. In most countries, there are strict criteria, governmental laws, and regulations for the performance of any kind of reproductive donation. A recent study conducted in the Netherlands reported that a typical donor was very well-educated, feminine, and a fellow who has agreed to make donations for altruistic reasons (13). None of the women who participated in the study reported that the earnings from the financial direction motivated them and did not show them as the reasons for the donation (13). Another multi-centre study reported that the majority of donors undergo the donation programme for altruistic reasons; however, the sociodemographics and motivation of donors vary hugely depending on the country of donation (9). On the contrary, financial motivation, especially as the compensation increases, has been reported to be the main drive of oocyte donors in the United States of America (USA) (9). Similarly, in this study, 70% of the participants clearly stated that financial gains were their main reason for going through the donation programme.

One of the main reasons of the inconsistency of these motivations may be due to the differences in the socioeconomic status of the local donors in North Cyprus compared with other European countries where similar studies were conducted. This introduces critical ethical and psychological issues in countries like North Cyprus, which is considered to be a third world country. The greatest difficulties in such countries are the lack of robust, local, governmental regulations and/or enforcement of existing regulations on ART centres carrying out oocyte donation procedures. Therefore, especially not having a set limit on the reimbursement makes it very tempting for potential donors. On the other hand, even though reimbursement is controversial, it has been proposed that it is not realistic for women to go through the donation programme just based on altruistic basis (9,17). Even women with altruistic motivation, inconveniences such as transport to the ART clinic and taking time off work, should have a small financial compensation to encourage them to go through the donation process (9). Previously published studies reported that these donors with altruistic motivation were usually married and well-educated (17-20), whereas donors who go through the donation programme due to financial gains have variable demographics and tend to be single and younger (21,22,23). The mean age of the oocyte donors in this study was lower than reported in other studies (19,23,24,25), and they were mostly single students. Therefore, the demographics of the donors in North Cyprus are different compared with other parts of Europe.

Due to the differences of laws and regulations in each country, the results of these kinds of studies vary significantly (26). Throughout Europe and the USA, the legislation on anonymity or reimbursement vary reflecting the different drives of involvement in the oocyte donation programmes. There is a need of more studies to investigate the motivation and counselling in different countries to obtain a better insight to avoid donor exploitation. Our results showed that the donors were being informed selectively about certain risks and they gained knowledge with multiple donation cycles by experience. All donors were informed about OHSS because this is a very serious and life-threatening condition. However, fewer donors knew about the risk of (multiple/ectopic) pregnancy following unprotected sexual intercourse during the treatment cycle. Both of these are very serious conditions with short-term effects and the clinic would be in huge distress in the event that they happened. However, information provided about long-term medical risks, which might frighten the potential donor, were not provided sufficiently. Even though donors with a previous donation history were generally more informed, they were equally as uninformed as a first-time donor about the possible long-term consequences of donation. Therefore, a standardised counselling protocol is missing. Furthermore, there is a critical risk of donor exploitation, especially in third world countries where donors tend to have lower socioeconomic status and lower self-esteem. Therefore, it is advised to set up large-scale longitudinal studies to establish sufficient counselling services and set an amount for compensation for each country without the risk of donor exploitation.
